# Reduced glutathione level in the aqueous humor of patients with primary open-angle glaucoma and normal-tension glaucoma

**DOI:** 10.1038/s41514-023-00124-2

**Published:** 2023-11-21

**Authors:** Kota Sato, Daisuke Saigusa, Taiki Kokubun, Amane Fujioka, Qiwei Feng, Ritsumi Saito, Akira Uruno, Naomi Matsukawa, Michiko Ohno-Oishi, Hiroshi Kunikata, Yu Yokoyama, Masayuki Yasuda, Noriko Himori, Kazuko Omodaka, Satoru Tsuda, Shigeto Maekawa, Masayuki Yamamoto, Toru Nakazawa

**Affiliations:** 1 Department of Ophthalmology, Tohoku University Graduate School of Medicine, Sendai, Miyagi Japan; 2 Department of Ophthalmic Imaging and Information Analytics, Tohoku University Graduate School of Medicine, Sendai, Miyagi Japan; 3 Laboratory of Biomedical and Analytical Sciences, Faculty of Pharma-Science, Teikyo University, Tokyo, Japan; 4 Department of Integrative Genomics, Tohoku Medical Megabank Organization, Tohoku University, Sendai, Miyagi Japan; 5 Medical Biochemistry, Tohoku University School of Medicine, Sendai, Miyagi Japan; 6 Department of Aging Vision Healthcare, Tohoku University Graduate School of Medicine, Sendai, Miyagi Japan; 7 Department of Retinal Disease Control, Tohoku University Graduate School of Medicine, Sendai, Miyagi Japan; 8 Department of Advanced Ophthalmic Medicine, Tohoku University Graduate School of Medicine, Sendai, Miyagi Japan; 9 Department of Collaborative Program for Ophthalmic Drug Discovery, Tohoku University Graduate School of Medicine, Sendai, Miyagi Japan

**Keywords:** Biomarkers, Glaucoma

## Abstract

Glaucoma is a leading cause of blindness worldwide in older people. Profiling the aqueous humor, including the metabolites it contains, is useful to understand physiological and pathological conditions in the eye. In the current study, we used mass spectrometry (MS) to characterize the aqueous humor metabolomic profile and biological features of patients with glaucoma. Aqueous humor samples were collected during trabeculectomy surgery or cataract surgery and analyzed with global metabolomics. We included 40 patients with glaucoma (32 with POAG, 8 with NTG) and 37 control subjects in a discovery study. VIP analysis revealed five metabolites that were elevated and three metabolites that were reduced in the glaucoma patients. The identified metabolomic profile had an area under the receiver operating characteristic curve of 0.953. Among eight selected metabolites, the glutathione level was significantly decreased in association with visual field defects. Moreover, in a validation study to confirm the reproducibility of our findings, the glutathione level was reduced in NTG and POAG patients compared with a cataract control group. Our findings demonstrate that aqueous humor profiling can help to diagnose glaucoma and that various aqueous humor metabolites are correlated with clinical parameters in glaucoma patients. In addition, glutathione is clearly reduced in the aqueous humor of glaucoma patients with both IOP-dependent and IOP-independent disease subtypes. These findings indicate that antioxidant agents in the aqueous humor reflect glaucomatous optic nerve damage and that excessive oxidative stress may be involved in the pathogenesis of glaucoma.

## Introduction

Glaucoma has become the most common cause of irreversible blindness worldwide^1^. The prevalence of glaucoma is increasing in aging populations and has reached ~5% in people aged 40–80 years^2^. Glaucoma is characterized by the progressive loss of retinal ganglion cells (RGCs), resulting in characteristic visual field defects. One of the most important risk factors for glaucoma is elevated intraocular pressure (IOP), which promotes mechanical stress in RGCs and the lamina cribrosa, blockading axonal transport and causing axonal degeneration^3,4^. Thus, all glaucoma treatments, both pharmacological and surgical, focus on lowering IOP. Nevertheless, even with successful IOP reduction, some glaucoma patients still undergo progressive visual field loss^5^, suggesting that various IOP-independent factors contribute to glaucoma. Animal experiments and studies of human subjects have shown that IOP-independent factors include oxidative stress, glutamate excitotoxicity, and impaired retinal blood flow^6–8^. These risk factors have also been reported to be augmented with age^9–12^.

Genetic factors are also associated with glaucoma onset. Genome-wide association studies have demonstrated that CDKN2B-AS1 is one of the most important non-population-specific genes associated with glaucoma^13,14^. CDKN2B-AS1 regulates transcriptional levels of CDKN2A, which is a marker of aging. Our previous work showed that CDKN2A expression is elevated in excitotoxicity-damaged RGCs and that CDKN2A knockdown ameliorates RGC death in mice^15^. Additionally, staining for SA-βgal was positive in mice with ischemic injury caused by IOP and in the RGCs of human subjects with POAG^16,17^. Moreover, depleting senescent retinal cells has been shown to protect the RGCs in experimental glaucoma^18^. Taken together, these studies indicate that aging is highly involved with the pathology of glaucoma.

Despite much research, the molecular mechanisms and pathologies underlying the onset and progress of glaucoma are still unclear. Approaches to past research include analyzing the aqueous humor, which is thought to be a useful way to discover specific molecules that contribute to glaucoma pathophysiology, and a proteomic approach, which has been used to identify proteins that are candidates for making prognoses in glaucoma pathogenesis. Previous studies have demonstrated that the aqueous humor of glaucoma patients shows alterations in the levels of certain proteins, such as those that contribute to oxidative damage, mitochondrial damage, neural degeneration, apoptosis, immune responses, and the complement cascade^19–22^. Metabolomics techniques have also been used to investigate many physiological and pathological conditions, including glaucoma. Previous work has used these techniques to identify various metabolites that are altered in the aqueous humor of glaucoma patients^23–27^. However, previous studies have not resolved certain questions: i) whether alterations in the identified proteins and metabolites are affected by eye drops, ii) how these changes in proteins and metabolites are affected by glaucoma phenotype, and iii) whether normal-tension glaucoma shows protein and metabolite changes in the same way as POAG. These questions must be answered to understand the pathological mechanisms underlying glaucoma.

In this study, we used non-targeted metabolomics to identify changes in the aqueous humor of glaucoma patients. The aims of this study were to identify biomarkers that precisely reflect visual field defects and the loss of RGCs in human subjects, and to identify IOP-independent biomarkers of glaucoma in a validation study based on targeted metabolomics.

## Results

### Global metabolomics

We started by selecting three assays, including HILICpos, HILICneg, and C18pos, based on data quality as measured with global metabolomics (G-Met) in a previous report published by us^28^. The detected features after peak picking were 5436, 5802, and 17,579 for HILICpos, HILICneg, and C18pos, respectively. After a normalization procedure with ProgenesisQI, which we also previously described, we determined ions estimated to have derived from the eye drop-delivered drugs, as well as the metabolites of these drugs and their additives (Supplementary Table 1). The G-Met analyses of human aqueous humor samples identified 31, 13, and 13 eye drop-derived ions in the HILICpos, HILICneg, and C18pos assays, respectively. We used 1489, 1349, and 2705 features for HILICpos, HILICneg, and C18pos, respectively, in the subsequent multivariate analyses.

**Fig. 1:**
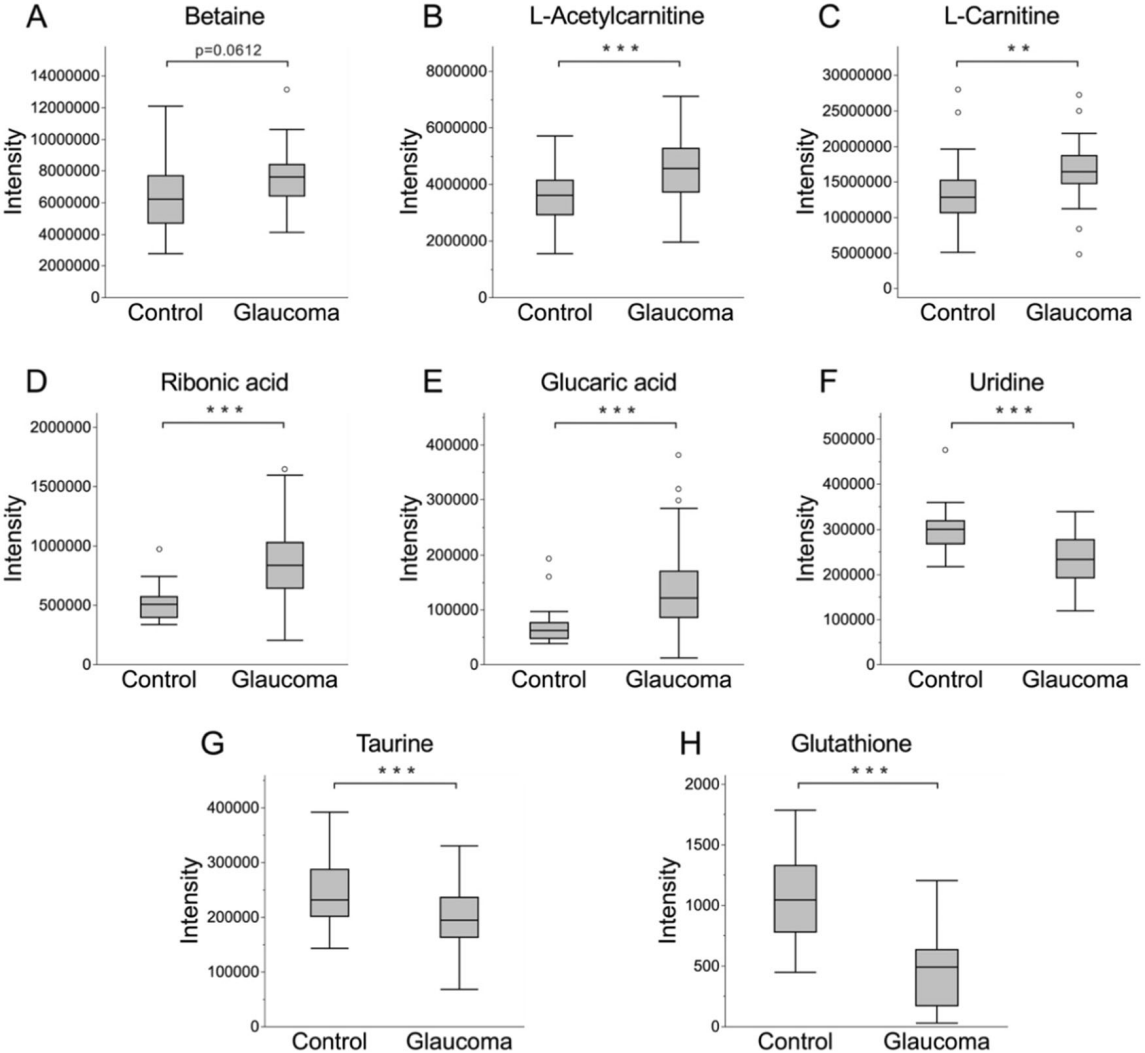
Boxplots of the eight most significant metabolites from a VIP analysis comparing control and glaucoma subjects in discovery study. Graphs showing metabolite intensity of betaine (**A**), L-acetylcarnitine (**B**), L-carnitine (**C**), ribonic acid (**D**), glucaric acid (**E**), uridine (**F**), taurine (**G**), and glutathione (**H**). This statistical analysis used the Student *t* test. **P < 0.01, ***P < 0.001.

**Fig. 2:**
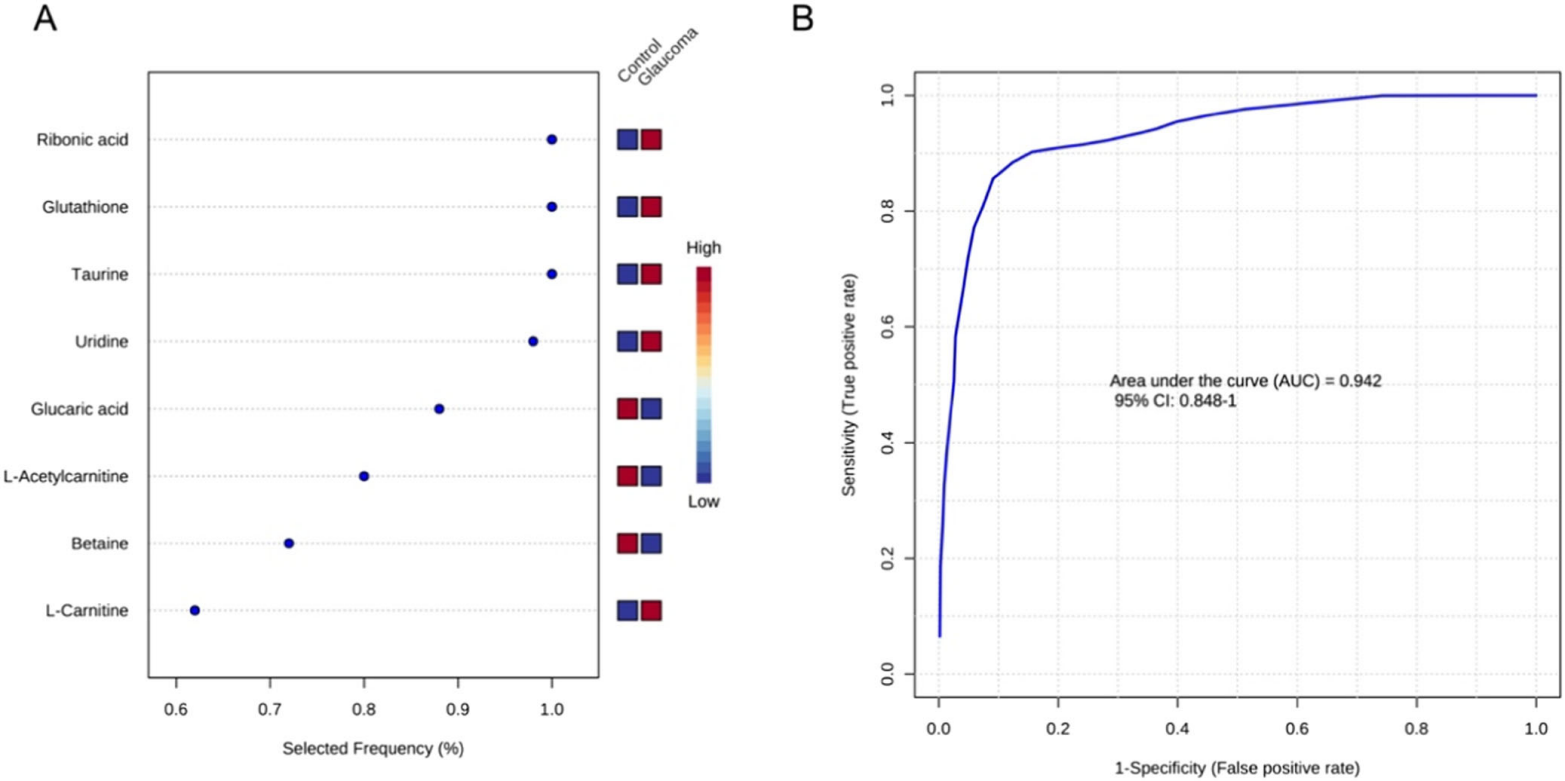
Classification of the glaucoma using a model including metabolites from the aqueous humor via Metaboanalyst 5.0. The eight significant metabolites ranked based on their frequencies of being selected during cross-validation (**A**) and receiver operating characteristic (ROC) curve analysis (**B**) are shown.

The principal component analysis (PCA) score plot for HILICpos, HILICneg, and C18pos showed that the distribution for study quality control samples, which mixed all aqueous humor samples, was observed in the center, indicating that the analysis had high quality (Supplementary Fig. 1). Then, we extracted 66, 63, and 32 features for HILICpos, HILICneg, and C18pos, respectively, that contributed to glaucoma with an orthogonal partial least square-discriminant analysis (OPLS-DA) S-plot based on the following high and low values for glaucoma: CV > 0.42 and CV < −0.5 for HILICpos, CV > 0.42 and CV < −0.45 for HILICneg, and CV > 0.52 or p[1] > 0.06 and CV < −0.7 or p[1] < −0.08 for C18pos (Supplementary Table 2). Finally, 11, 24, and 16 features for HILICpos, HILICneg, and C18pos, respectively, were selected according to VIP score > 1.5 (Supplementary Table 3). The list of identified metabolites in the three assays is shown in Tables 1 and 2. Five metabolites (betaine, L-acetylcarnitine, L-carnitine, ribonic acid, and glucaric acid) increased and three metabolites (uridine, taurine, and glutathione) significantly decreased in the aqueous humor of glaucoma patients (Fig. 1). The analysis indicated that N,N-Diethylethanolamine was elevated in the aqueous humor of glaucoma patients and thus a biomarker, but we suspected that it was derived from oxybuprocaine (Supplementary Fig. 2). The Human Metabolome Database states that N,N-diethylethanolamine is used in the production of a variety of chemical commodities, such as the local anesthetic procaine. Both glaucoma and control group patients were treated with oxybuprocaine (brand name Benoxil) eye drops before surgery, making it highly likely that the presence of this metabolite in the aqueous humor samples was due to the eye drops. Thus, we excluded N,N-diethylethanolamine from further analysis. To further evaluate the effect of eye drops on the metabolomic profile of the aqueous humor, we performed association analyses with and without eye drops (Supplementary Table 4). Five metabolites (L-acetylcarnitine, L-carnitine, ribonic acid, glucaric acid, and taurine) were significantly increased in a group of patients that received acetazolamide oral administration (Supplementary Fig. 3), suggesting that these metabolites may be a feature of glaucoma with uncontrollable IOP. In addition, we generated an ROC curve for the support vector machines (SVMs) using the identified metabolites. The area under the ROC curve was 0.953% and the 95% confidence interval was 0.872–1 for the eight identified metabolites that were included in a model predictor (Fig. 2). These findings demonstrate that the aqueous humor profile of our identified metabolites reflects the characteristics of eyes with glaucoma and that these metabolites have the potential to support the diagnosis of glaucoma.

**Table 1:** List of metabolites that increased in the glaucomatous aqueous humor.

Mode	*m/z*	*t*_R_	Compounds	Formula	Adduct	*m/z*	delta	ID	VIP score
	(Detected)					(Theoretical)	ppm		
HILICpos	118.0858	3.83	Betaine	C5H11NO2	M+H	118.0863	4.2	HMDB0000043	3.2
HILICpos	204.1210	3.92	L-Acetylcarnitine	C9H17NO4	M+H	204.123	9.8	HMDB0000201	4.5
HILICpos	162.1110	4.49	L-Carnitine	C7H15NO3	M+H	162.1125	9.3	HMDB0000062	7.9
HILICneg	165.0409	3.98	Ribonic acid	C5H10O6	M-H	165.0405	−2.4	HMDB0000867	4.9
HILICneg	209.0308	5.27	Glucaric acid	C6H10O8	M-H	209.0303	−2.4	HMDB0000663	2.1

**Table 2:** List of metabolites that decreased in the glaucomatous aqueous humor.

Mode	*m/z*	*t*_R_	Compounds	Formula	Adduct	*m/z*	delta	ID	VIP score
	(Detected)					(Theoretical)	ppm		
C18pos	308.0908	1.58	Glutathione	C10H17N3O6S	M+H	308.0911	1.0	HMDB0000125	1.8
HILICneg	243.0626	3.30	Uridine	C9H12N2O6	M-H	243.0623	−1.2	HMDB0000296	2.4
HILICneg	124.0075	4.58	Taurine	C2H7NO3S	M-H	124.0074	−0.8	HMDB0000251	1.6

### Associations between metabolites in the aqueous humor of glaucoma patients and with clinical parameters

We analyzed the associations between the identified metabolites with Spearman’s rank correlation coefficient analysis. Glucaric acid and ribonic acid had the most significant positive correlation with each other, and there were also highly positive correlations between L-acetylcarnitine and L-carnitine, and between taurine and glutathione (Table 3). Next, we analyzed the associations of these metabolites with clinical parameters in glaucoma patients. The aqueous humor level of glucaric acid and taurine was positively associated with preoperative IOP, and L-carnitine was positively associated with corneal thickness (Table 4 and Supplementary Fig. 4). To clarify the involvement of glucaric acid in changes to trabecular meshwork (TM) cells that contribute to IOP elevation, we performed a series of in vitro human trabecular meshwork cell (HTMCs) experiments. First, to elucidate the effect of glucaric acid on HTMCs contraction, we measured changes in gel diameter every 24 h for 7 days. We used TGF-β1 as a positive control because it is known to induce contraction of TM cells^29^. The gel diameter of the TGF-β1-treated HTMCs was significantly lower than the negative controls, but the HTMCs cultured with glucaric acid did not significantly differ from the negative controls (Supplementary Fig. 5A). Next, we evaluated the expression levels of αSMA, which has been reported to be associated with cell contractility; it is embedded in the actin cytoskeleton and improves cell contractile strength. Glucaric acid-treated HTMCs had a similar expression level of αSMA as negative controls (Supplementary Fig. 5B). These results show that glucaric acid was not the cause of TM change and may not cause the elevation of IOP. In other words, the high levels of glucaric acid in the aqueous humor may result from stimulation by elevated IOP.

**Table 3:** Correlation coefficient between metabolites in the aqueous humor.

**Table 4:** Correlation coefficient between clinical parameters and metabolites in the aqueous humor. (*P < 0.05, **P < 0.01).

	Age	Preoperative IOP	Corneal thickness	MD value	Axial length
Betaine	−0.1861	0.2591	−0.0219	0.0341	0.0111
L-Acetylcarnitine	0.0642	0.0757	0.3055	−0.0254	−0.1362
L-Carnitine	−0.0125	0.1488	0.3702*	−0.0170	−0.1745
Ribonic acid	−0.1873	0.2544	−0.0736	−0.1278	0.2886
Glucaric acid	−0.1235	0.4456**	−0.0899	−0.1227	0.2714
Uridine	−0.2080	−0.0062	0.2132	−0.2842	−0.1469
Taurine	0.1924	0.4710**	0.2411	−0.0707	−0.2554
Glutathione	0.1722	−0.0534	0.2142	0.0352	−0.1750

**Fig. 3:**
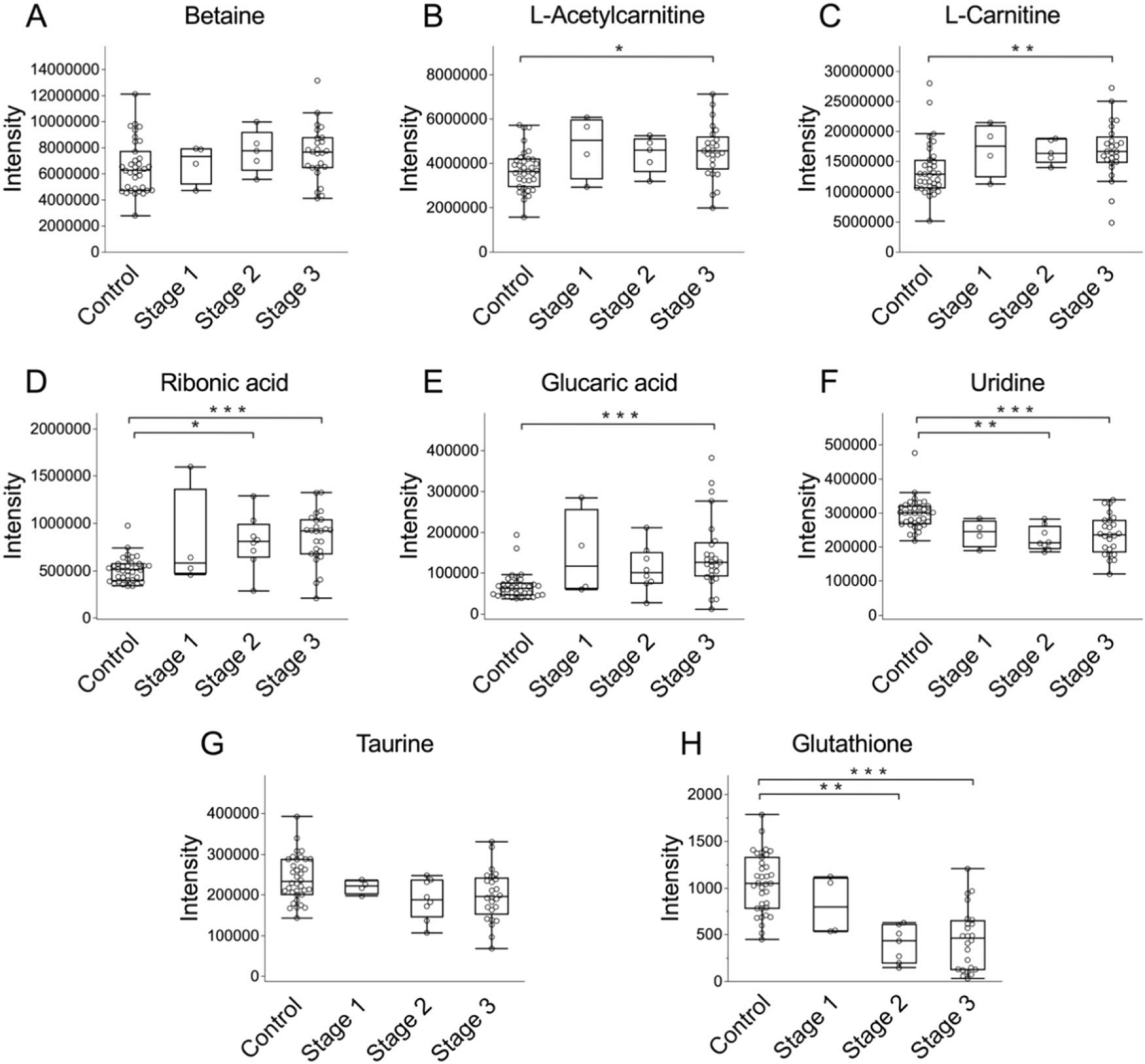
The relationship between the levels of eight selected metabolites and the visual field in glaucoma patients. Boxplots showing metabolite intensity of betaine (**A**), L-acetylcarnitine (**B**), L-carnitine (**C**), ribonic acid (**D**), glucaric acid (**E**), uridine (**F**), taurine (**G**), and glutathione (**H**) in the aqueous humor of control subjects and patients with glaucoma stage 1, stage 2, and stage 3. The statistical analysis used Dunn’s multiple comparison test (*P < 0.05, **P < 0.01, ***P < 0.001).

Next, to assess the identified metabolites and clinical phenotypes in further detail, we analyzed their association with visual field grades, because visual field loss is one of the most important criteria for intervention. Glaucoma patients were classified into three groups, either Stage 1 (MD > −6 dB), Stage 2 (−12 < MD < −6 dB) or Stage 3 (MD < −12 dB), according to mean deviation (MD) of the Humphrey field analyzer. Among the 8 identified metabolites, the levels of L-acetylcarnitine, L-carnitine, ribonic acid, and glucaric acid tended to increase from stage 1 and became significantly higher in stage 3 when compared to controls (Fig. 3A–E). Uridine and glutathione levels in the aqueous humor of POAG patients also tended to decrease from stage 1 and became significantly lower in stage 2 and 3 when compared to controls (Fig. 3F–H). These findings suggest that these metabolites may have the potential to be valuable surrogate markers of glaucomatous neurodegeneration.

### Reduced glutathione levels in the aqueous humor of NTG and POAG patients in the validation study

**Fig. 4:**
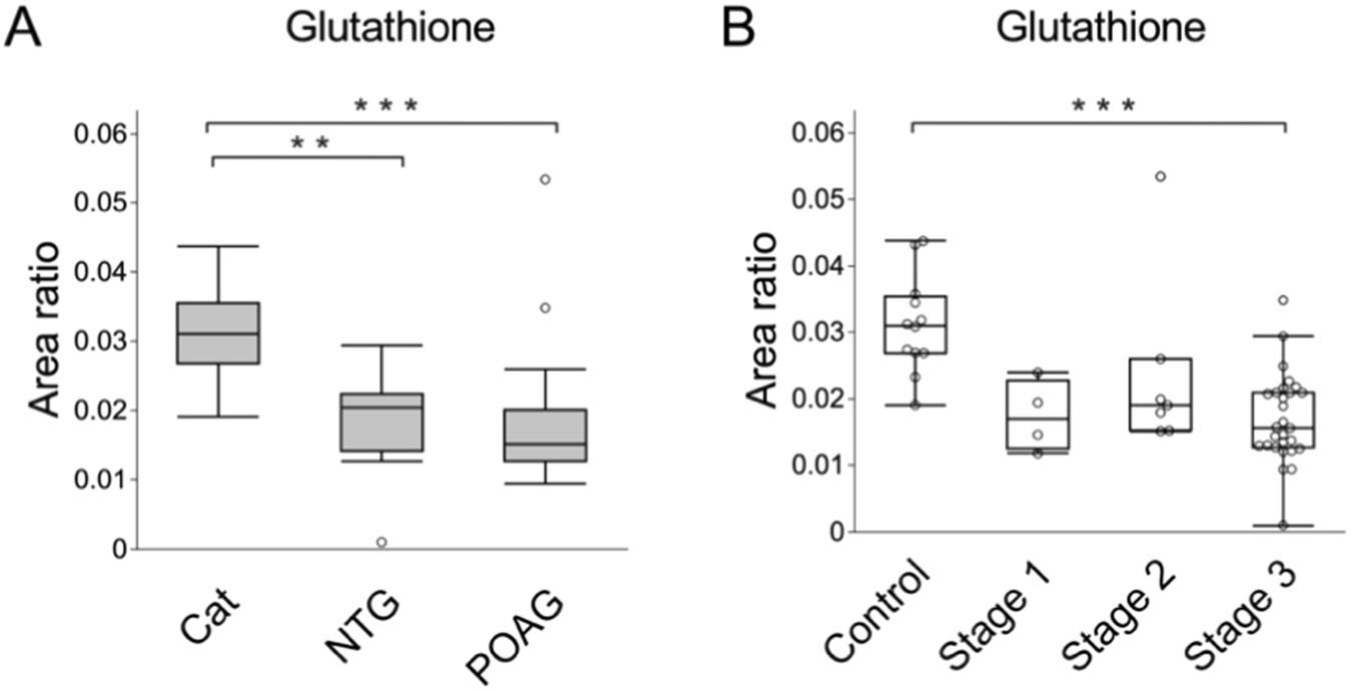
Glutathione levels and their association with the visual field in the validation study. **A** Glutathione levels were measured in the aqueous humor of patients with cataract, NTG, and POAG with targeted metabolomics. The statistical analysis used the Steel-Dwass test. **P < 0.01, ***P < 0.001. **B** Boxplots showing metabolite intensity of glutathione in the aqueous humor of control subjects and patients with glaucoma stage 1, stage 2, and stage 3. The statistical analysis was performed with Dunn’s multiple comparison test (***P < 0.001).

Our above analysis indicated that glutathione levels gradually but clearly dropped with visual field loss. We also confirmed that glutathione levels were reduced in NTG patients because most of the glaucoma population in the discovery study (shown in Fig. 3) comprised patients with POAG. As a validation study, we obtained aqueous humor samples from a separate population of NTG and POAG patients, with cataract patients as a control group. Targeted liquid chromatography/mass spectrometry (LC/MS) analysis showed that glutathione levels were not only significantly reduced in NTG, but also in POAG (Fig. 4A). Moreover, the NTG and POAG patients in the validation study population also had a significant reduction in patients with stage 3 visual field loss (Fig. 4B). These findings suggest that a reduction in the antioxidant properties of the aqueous humor is a common feature in IOP-dependent and IOP-independent glaucoma patients.

## Discussion

This study produced a metabolomic profile of the aqueous humor in glaucoma patients based on a new, more precise method that combined non-targeted global metabolomics, a statistical analysis, and the exclusion of eye drop-derived metabolites. We found several metabolites that were associated with clinical parameters in glaucoma and had high accuracy to distinguish glaucoma. We also found that antioxidant agents such as taurine and glutathione were selected from more than five thousand features and were significantly reduced in the aqueous humor of glaucoma patients. Finally, among our eight selected metabolites, a reduction in glutathione was most reflective of the grade of visual field loss in NTG and POAG patients.

Reinforcing previous findings^27^, our study found that L-carnitine and L-acetylcarnitine were elevated in the glaucomatous aqueous humor. Both of these carnitine metabolites are known as serum biomarkers of preeclampsia and have been shown to increase under hypoxic conditions in a cultured placental choriocarcinoma cell line (the BeWo line)^30^. Previously, we revealed that optic nerve blood flow is reduced in glaucoma patients, even patients with preperimetric glaucoma, i.e., before visual field loss occurs^8^. Together, these findings suggest that L-carnitine and L-acetylcarnitine elevation in the aqueous humor of glaucoma patients may be due to chronic hypoxic conditions in the eye. During hypoxia, the metabolic flow of glucose changes to reduce acetyl-CoA and promote lactate production. Carnitine plays a role in transporting fatty acids across the inner mitochondrial membrane for β-oxidation. Thus, the elevation of carnitine metabolites we observed in the aqueous humor is likely to promote ATP production through β-oxidation to compensate for glycolysis. A recent study showed that glucose metabolism was dysregulated and that pyruvate supplementation could prevent neurodegeneration in an animal model of glaucoma^31^, suggesting that ATP production from glycolysis is disrupted, and that other energy fuel pathways, such as β-oxidation, may be augmented to compensate for the reduction in energy metabolism. In addition, our previous work also showed that retinal L-acetylcarnitine was elevated after optic nerve crush in mice^32^. In fact, L-carnitine and L-acetylcarnitine are established mitochondrial biomarkers used to screen neonates for a series of genetic disorders affecting fatty acid oxidation^33^. Thus, elevated carnitine metabolites may reflect a neuroprotective response to compensate for metabolic dysfunction, resulting in RGC death.

We also detected a reduction of taurine in the aqueous humor of glaucoma patients, which is consistent with previous findings^34^. The functions of taurine include the improvement of mitochondria-related diseases such as mitochondrial myopathy, encephalopathy, lactic acidosis, and stroke-like episodes and aging-related diseases^35^. Taurine reduction has also been reported in the corneas of aged mice^36^. Recently, it has been reported that systemic taurine reduction was found in aging animals, including humans, and that a taurine supplement increased healthy lifespan^37^. We also found that glutathione was reduced in the aqueous humor of glaucoma patients in the current study. Glutathione and taurine are well known to function as antioxidants^38,39^. Additionally, we previously reported that glutathione levels in the leukocytes of glaucoma patients were reduced compared to control subjects^40^. Finally, we previously used an animal experiment to show that RGC loss in Nrf2 knockout mice was much faster after optic nerve damage was induced by crushing the nerve^41^. These findings suggest that a decrease in anti-oxidative agents is a risk factor for glaucoma onset and progression.

In conclusion, our non-targeted metabolomics approach identified several molecules, such as carnitine derivatives, and antioxidant agents, such as taurine and glutathione. These metabolites may be involved with the development of glaucomatous pathophysiology. To determine the underlying mechanisms in detail, further study will be needed. A key point of our study is our finding of a reduction of glutathione in NTG patients, suggesting that excessive oxidative stress may contribute to optic nerve damage and RGC loss independently of high IOP in glaucoma.

## Methods

### Materials

LC/MS-grade methanol, acetonitrile and ammonium hydroxide were purchased from Kanto Chemical (Tokyo, Japan). Ammonium bicarbonate (1 mol/L) was purchased from Cell Science & Technology Inst., Inc. (Miyagi, Japan). Ammonium formate (1 mol/L) and LC/MS-grade formic acid were purchased from Fujifilm Wako Chemical Industries (Osaka, Japan). Glutathione (GSH)-^13^C_2_, ^15^N was purchased from Taiyo Nissan Corp. (Tokyo, Japan) and used as internal standard (IS). All drugs were obtained from Senju Pharmaceutical Co., Ltd. (Osaka, Japan), Santen Pharmaceutical Co., Ltd. (Osaka, Japan), Pfizer Japan Inc. (Tokyo, Japan), Kowa Co., Ltd. (Aichi, Japan) or Novartis Pharma K.K. (Tokyo, Japan).

### Human subjects

Human subjects were included in this study. The research was conducted according to the tenets of the Declaration of Helsinki and was approved by the Tohoku University Hospital Review Board. All included participants provided written informed consent and were recruited prospectively.

### Aqueous humor samples from glaucoma patients

Samples of human aqueous humor were obtained from glaucoma patients at the Department of Ophthalmology, Tohoku University Hospital. The research ethics committee of the School of Medicine, Tohoku University, approved this study, which was conducted in accordance with the ethical guidelines of the 1975 Declaration of Helsinki. All the patients provided written informed consent for the use of clinical samples. The aqueous humor samples were stored at −80 °C until the analysis. Demographic data are shown in Table 5.

**Table 5:** Demographic data for the discovery study.

	OAG	Control	*P* value
No. of eyes	40	37	
Age (years)^†^	64.3 ± 11.6	66.6 ± 7.1	0.2928
Laterality (right:left)^‡^	17:23	23:14	0.1113
Sex (male:female)^‡^	21:19	17:20	0.6505
IOP (mmHg)^†^	18.1 ± 6.2	13.7 ± 3.3	0.0003
BCVA (logMAR)^†^	0.39 ± 0.57	0.58 ± 0.42	0.1023
Axial length (mm)^†^	24.9 ± 1.6	24.0 ± 1.6	0.0153
Glaucoma medications, *n* (%)			
Prostaglandin analogs	39 (97.5)	0 (0)	
β-blockers	35 (87.5)	0 (0)	
Carbonic anhydrase inhibitors (eye drop)	31 (77.5)	0 (0)	
Carbonic anhydrase inhibitors (dose)	15 (37.5)	0 (0)	
α-2 stimulates	32 (80.0)	0 (0)	
Rho-kinase inhibitors	15 (37.5)	0 (0)	

^†^*P* values and ^‡^*P* values are calculated between OAG and Control groups using the Student’s *t* test and Fisher’s exact test, respectively.

### Sample preparation for global metabolomics

Each sample (50 μl) was placed in a well on a 96-well plate. The sample preparation procedure was performed by a robotic system (Hamilton, Reno, NV) as described in previous reports^28^.

### Global metabolomics

G-Met analyses of the aqueous humor samples from control subjects (total, *n* = 37: cataract, *n* = 12; epiretinal membrane, *n* = 8; macular hole, *n* = 17) and glaucoma patients (total, *n* = 40: normal-tension glaucoma, *n* = 8; primary open-angle glaucoma, *n* = 32) were performed with ultra-high-performance liquid chromatography quadruple time-of-flight mass spectrometry (UHPLC-QTOF/MS; Synapt G2-Si QTOF MS, Waters Corp., Manchester, UK) with reverse-phase (C18) column (Acquity HSS T3; 150 mm × 2.1 mm i.d., 1.8 μm particle size; Waters) positive (C18pos) and negative (C18neg) ion modes and high-performance liquid chromatography Fourier transform mass spectrometry (LC-FTMS; Q Exactive Orbitrap MS, Thermo Fisher Scientific, San Jose, CA) with a normal-phase (hydrophilic interaction chromatography; HILIC) column (ZIC-pHILIC; 100 mm × 2.1 mm i.d., 5 μm particle size; Sequant, Darmstadt, Germany) with positive (HILICpos) and negative (HILICneg) ion modes. Details of the settings used for UHPLC-QTOF/MS and LC-FTMS were previously described^28^.

### Data processing

All data obtained from the four assays with the two systems were processed with software (Progenesis QI 3.0.3.0, Nonlinear Dynamic, Newcastle, UK) for peak picking, alignment, and normalization to produce peak intensities for retention time (*t*_R_) and mass-to-charge ratio (*m/z*) data pairs. First, the features (identified by *t*_R__*m/z*) were selected based on both their coefficient of variation (CV) and the inverse correlation of the dilution fold and peak intensity. This used our normalization process to exclude contaminants, as previously described^28^. Then, ions estimated to be derived from eye drop-delivered drugs or their metabolites, or from additives in the eye drops, were excluded from the features manually. Patients reported, in interview forms, using the following drugs: brinzolamide, latanoprost, ripasudil, travoprost, carteolol, isopropyl unoprostone, tafluprost, timolol, bunazosin, bimatoprost, brimonidine, bromfenac, gatifloxacin, levofloxacin, phenylephrine and tropicamide. After excluding drug-derived metabolites, the remaining features were examined for biomarkers of glaucoma with multivariate analysis, including a PCA and an OPLS-DA, with EZinfo software (Waters). Next, the extracted features were identified with an analysis based on UHPLC-FTMS (Orbitrap Fusion, Thermo Fisher Scientific). The MS spectra, MS/MS spectra, isotopic ratios, mass accuracy, and chromatographic *t*_R_ were matched with compounds in the Human Metabolome Database (HMDB, http://www.hmdb.ca), the LIPID MAPS Structure Database (https://www.lipidmaps.org/data/structure/index.php), mzCloud (https://www.mzcloud.org) and ChemSpider (http://www.chemspider.com). *P* values were calculated using the Student’s *t* test using JMP software. The LC-MS data included some missing values, which were handled as follows: if the missing values accounted for more than 20% of samples, the metabolite was excluded from the analysis. If the missing values accounted for less than 20%, only the missing values were excluded.

### Receiver operating characteristic curve analysis

The receiver operator characteristic (ROC) curve and the area under the curve were calculated with MetaboAnalyst 5.0 (http://www.metaboanalyst.ca) software. Missing values were replaced by 1/5 of the minimum positive value for each variable, and auto-scaling was selected as the data scaling option. Support vector machines (SVMs) were used for the ROC curve analysis.

### Sample preparation for UHPLC-MS/MS

The methanol (1.2 mL) containing the IS solution (10 µg/mL, GSH-^13^C_2_, ^15^N for negative ion mode (Neg)) was added to the cell-cultured plate and maintained for 2 min. Then, the solution was transferred 1.5-mL tube and stored at −80 °C until analysis. The sample was homogenized for 30 s by mixer, and sonication for 10 min on the ultra-sonic bath. After centrifuged at 16,400 × *g* for 20 min at 4 °C, the total of 0.7 mL of supernatant was transferred to another 1.5-mL tube. Finally, aliquot (3-µL) of the deproteinized sample was analyzed by means of an ultra high-performance liquid chromatography triple quadrupole mass spectrometry (UHPLC-MS/MS). Demographic data are shown in Table 6.

**Table 6:** Demographic data for glutathione measurement in the validation study.

	Cat	NTG	POAG	P value		
No. of eyes	12	12	25			
Age (years)^†^	71.1 ± 6.2	69.8 ± 11.2	68.7 ± 6.6	Cat	vs NTG	0.8759
					vs POAG	0.5890
Laterality (right: left)^‡^	3:9	6:6	10:15			0.4726
Sex (male: female)^‡^	5:7	8:4	16:9			0.3865

^†^*P* values and ‡ *P* values are calculated using the Dunnett test and Fisher’s exact test, respectively.

### UHPLC-MS/MS analysis

The UHPLC-MS/MS analysis was performed on Acquity™ Ultra Performance LC I-class system equipped with a binary solvent manager, a sample manager, and a column heater (Waters Corp. Milford, UK) interfaced to a Waters Xevo TQ-S MS/MS system equipped with ESI operated in Neg. The MS/MS was performed using the multiple reaction monitoring (MRM) mode with a scan time of 0.005 s for each compound. The transitions of the precursor ion to the product ion, cone voltage (V), and collision energy (eV) were listed (Supplementary Table 5). The capillary voltage was 2.5 kV, and the cone voltage was 100 V. The source offset and temperature were set at 50 V and 150 °C, respectively, with a cone gas flow rate of 150 L/h. The desolvation temperature was set at 450 °C, and the desolvation gas flow, collision gas flow, and nebulization gas flow were set to 1000 L/h, 0.15 min/mL, and 7.00 bar, respectively. Both the cone gas and the nebulization gas were nitrogen. LC separation, which followed the previously reported procedure^28^, was performed using a normal-phase (hydrophilic interaction chromatography, HILIC) column (ZIC-pHILIC; 100 mm × 2.1 mm i.d., 5 µm particle size; Sequant, Darmstadt, Germany) with a gradient elution of solvent A (10 mmol/L ammonium bicarbonate in water, pH 9.2) and solvent B (acetonitrile) at 300 µL/min. Solvent A was prepared by mixing 99 mL of Milli-Q (Millipore) water and 1 mL of 1 mol/L ammonium bicarbonate and was adjusted to a pH of 9.2 with 25% ammonium hydroxide (~0.1 mL). The initial condition was set at 99% B, linearly decreasing to 70% B from 0.5 min to 4.0 min, and 1% B from 4.0 min to 6.5 min, and was then maintained at 1% B for 2.5 min. Subsequently, the mobile phase was immediately returned to the initial conditions and maintained for 9 min until the end of the run. The oven temperature was 20 °C. The data were collected using the MassLynx v4.1 software (Waters Corp.) and the ratio of the peak area of analyte to the IS was analyzed by TraverseMS (Reifycs Inc., Tokyo, Japan).

### Human TM cell culture

Primary HTMCs were purchased from ScienCell Research Labs (Cat. # 6590; Carlsbad, CA, USA). The cells were maintained in TM cell medium (TMCM; Cat. # 6591; ScienCell Research Labs), which consists of a basal medium, 2% fetal bovine serum (FBS; Cat. # 0010), 1% TM cell growth supplement (TMCGS; Cat. # 6592), and 1% penicillin/streptomycin solution (P/S; Cat. # 0503). The TMCM was kept at 37 °C in 5% CO_2_ and passaged with the trypsin-EDTA method.

### Gel contraction

A collagen gel contraction assay was performed with the Collagen Gel Culturing Kit (Cat. # 638-00781; Fujifilm, Tokyo, Japan). HTMCs were trypsinized and resuspended in culture medium at a density of 2.2 × 10^6^ cells/ml. Cellmatrix Type I-A (3 mg/ml), 10 × MEM sterile culture medium (without NaHCO_3_), and sterile reconstitution buffer were mixed in an ice bath at a ratio of 8:1:1. Five identical 0.25 ml cell suspensions were prepared, to which we added TGF-β1 (20 μg/ml, Cat. # 240-B-002; R & D systems, Minneapolis, MN), glucaric acid (10 mM, Cat. # S4140; Sigma-Aldrich, St. Louis, MO, USA), or sterilized distilled water (2.5 μl) as a negative control. Next, 2.25 ml of kit mixture was added to each type of cell assay suspension and they were gently shaken. A 0.5 ml portion of each assay preparation was added to a well in a 24-well plate, and they were incubated at 37 °C in 5% CO_2_ for 1 h. After separating the gel from the bottom of the wells, the diameter of the collagen gel was measured using analysis software (BZ-X800E Analyzer; Keyence Corporation, Osaka, Japan) every 24 h from day 0 to day 7. The contraction effect of the HTMCs on the collagen gel was measured as the decrease in gel diameter compared to the control group.

### Western blotting

HTMCs were grown in the above-mentioned TMC medium in five 12-well plates at a density of 5 × 10^4^/ml for 24 h, and the medium was replaced with a new one, containing 1 μl of water (negative control), TGF-β1 (20 μg/ml) or glucaric acid (10 mM) for 10 min. Then, the cells were washed with PBS and lysed with PTTS buffer (0.5% Triton-100, 0.1% Tween-20, and 0.05% SDS) containing a protease inhibitor (Cat. # UD278492; Thermo Fisher Scientific, Waltham, MA, USA) and a phosphatase inhibitor (Cat. # 04906845001; Roche, Basilea, Switzerland) in an ice bath. The collected cell lysate was vortexed for 5 s and centrifuged at 12,000 rpm for 5 min at 4 °C. The supernatant was collected, and the protein concentration was determined with a BCA protein assay kit (Cat. # 23225; Thermo Fisher Scientific). Ten micrograms of each protein sample were separated into a 10% SDS-PAGE gel and transferred onto a polyvinylidene fluoride membrane. After blocking in 1% skim milk in Tw-PBS for 1 h at room temperature, the membrane was incubated with αSMA (dilution 1:2000; Cat. # A5228; Sigma-Aldrich) overnight at 4 °C. Next, the membrane was washed three times for 5 min per wash with Tw-PBS and incubated with goat anti-mouse IgG (dilution 1:5000; Cat. #12004159; BIO-RAD) for 1 h at room temperature. After washing three times for 5 min per wash with Tw-PBS, the signal was detected with ELC Prime Western Blotting Detection Reagent (Cat. # RPN2232; GE Healthcare, Piscataway, NJ, USA) and imaged using a chemiluminescence imager (ChemiDoc; Bio-Rad, Hercules, CA, USA) with Image Lab Software 3.0. Then, we reblotted the membrane with WB Stripping Solution (Cat. # 05364-55; Nacalai Tesque, Japan) and incubated it with β-actin (dilution 1:5000; Cat. # A5316; Sigma-Aldrich) as a loading control.

### Statistics

JMP Pro software version 15.0.0 (SAS Institute Inc., Cary, NC, USA) or GraphPad Prism7.05 was used for the statistical analysis. The statistical significance of differences between the glaucoma group and the control group was determined with the Student’s *t* test or Fisher’s exact test. The relationship between the metabolites and the visual field in glaucoma patients was statistically assessed with the Dunn’s multiple comparison test. The statistical analysis of glutathione levels of patients with cataract, NTG, and POAG was performed with the Steel-Dwass test. The effect of medication on the metabolomic profile of the human aqueous humor was statistically assessed with Student’s *t* test. The correlation coefficient analysis was performed with Spearman’s rank correlation coefficient analysis. The statistical significance of changes in HTMCs were calculated using Dunnett’s multiple test. *P* values < 0.05 were considered to be statistically significant.

### Reporting summary

Further information on research design is available in the Nature Research Reporting Summary linked to this article.

### Supplementary information

Supplementary Information

Supplementary Table 1

Supplementary Table 2

Supplementary Table 3

Supplementary Table 4

Supplementary Table 5

Reporting summary

## Data Availability

The source data for the findings of this study are available from the corresponding author upon reasonable request.
