# Author Correction: Reduced glutathione level in the aqueous humor of patients with primary open-angle glaucoma and normal-tension glaucoma

**DOI:** 10.1038/s41514-024-00137-5

**Published:** 2024-01-20

**Authors:** Kota Sato, Daisuke Saigusa, Taiki Kokubun, Amane Fujioka, Qiwei Feng, Ritsumi Saito, Akira Uruno, Naomi Matsukawa, Michiko Ohno-Oishi, Hiroshi Kunikata, Yu Yokoyama, Masayuki Yasuda, Noriko Himori, Kazuko Omodaka, Satoru Tsuda, Shigeto Maekawa, Masayuki Yamamoto, Toru Nakazawa

**Affiliations:** 1 Department of Ophthalmology, Tohoku University Graduate School of Medicine, Sendai, Miyagi Japan; 2 Department of Ophthalmic Imaging and Information Analytics, Tohoku University Graduate School of Medicine, Sendai, Miyagi Japan; 3 Laboratory of Biomedical and Analytical Sciences, Faculty of Pharma-Science, Teikyo University, Tokyo, Japan; 4 Department of Integrative Genomics, Tohoku Medical Megabank Organization, Tohoku University, Sendai, Miyagi Japan; 5 Medical Biochemistry, Tohoku University School of Medicine, Sendai, Miyagi Japan; 6 Department of Aging Vision Healthcare, Tohoku University Graduate School of Medicine, Sendai, Miyagi Japan; 7 Department of Retinal Disease Control, Tohoku University Graduate School of Medicine, Sendai, Miyagi Japan; 8 Department of Advanced Ophthalmic Medicine, Tohoku University Graduate School of Medicine, Sendai, Miyagi Japan; 9 Department of Collaborative Program for Ophthalmic Drug Discovery, Tohoku University Graduate School of Medicine, Sendai, Miyagi Japan

**Keywords:** Biomarkers, Glaucoma

Correction to: *npj Aging* 10.1038/s41514-023-00124-2, published online 21 November 2023

In this article the author information in Reference 26 was incorrect and should have appeared as shown below. The original article has been corrected.

Breda, J. B. et al. Metabolomic profiling of aqueous humor from glaucoma patients—the metabolomics in surgical ophthalmological patients (MISO) study. *Exp. Eye Res.* **201**, 108268 (2020).

