# Supplementary material for: Reduced glutathione level in the aqueous humor of patients with primary open-angle glaucoma and normal-tension glaucoma
Source: NPJ Aging. 2023 Nov 21;9(1):28. doi: 10.1038/s41514-023-00124-2 (PMC10663551; doi:10.1038/s41514-023-00124-2)
Supplement: Supplementary file 1 — Supplementary Information [file 41514_2023_124_MOESM1_ESM.pdf]

Supplementary Information

Reduced glutathione level in the aqueous humor of patients with primary open-angle glaucoma and normal-tension glaucoma

Kota Sato[1,2,a], Daisuke Saigusa[3,4,5,a], Taiki Kokubun[1,a], Amane Fujioka[1,a], Qiwei Feng[1,a], Ritsumi Saito[4,5], Akira Uruno[4,5], Naomi Matsukawa[4], Michiko Ohno-Oishi[1], Hiroshi Kunikata[1], Yu Yokoyama[1], Masayuki Yasuda[1], Noriko Himori[1,6], Kazuko Omodaka[1], Satoru Tsuda[1], Shigeto Maekawa[1], Masayuki Yamamoto[4,5], Toru Nakazawa[1,2,7,8 9,*]

([a]: Authors contributed equally to this work)

([*]: Corresponding author)

1 Department of Ophthalmology, Tohoku University Graduate School of Medicine, Miyagi, Japan

2 Department of Ophthalmic Imaging and Information Analytics, Tohoku University Graduate School of Medicine, Miyagi, Japan

3 Laboratory of Biomedical and Analytical Sciences, Faculty of Pharma-Science, Teikyo University, Tokyo, Japan

4 Department of Integrative Genomics, Tohoku Medical Megabank Organization, Tohoku University, Sendai, Miyagi, Japan.

5 Medical Biochemistry, Tohoku University School of Medicine, Sendai, Miyagi, Japan.

6 Department of Aging Vision Healthcare, Tohoku University Graduate School of

Medicine, Miyagi, Japan
7 Department of Retinal Disease Control, Tohoku University Graduate School of Medicine, Miyagi, Japan
8 Department of Advanced Ophthalmic Medicine, Tohoku University Graduate School of Medicine, Miyagi, Japan
9 Department of Collaborative Program for Ophthalmic Drug Discovery, Tohoku University Graduate School of Medicine, Miyagi, Japan

Correspondence to: Toru Nakazawa, Department of Ophthalmology, Tohoku University Graduate School of Medicine, 1-1, Seiryo, Aoba, Sendai, Miyagi 980-8574, Japan; Phone; +81 22 717 7294; Fax: +81 22 717 7298; E-mail: ntoru@oph.med.tohoku.ac.jp (T. Nakazawa).

Supplementary Figure 1

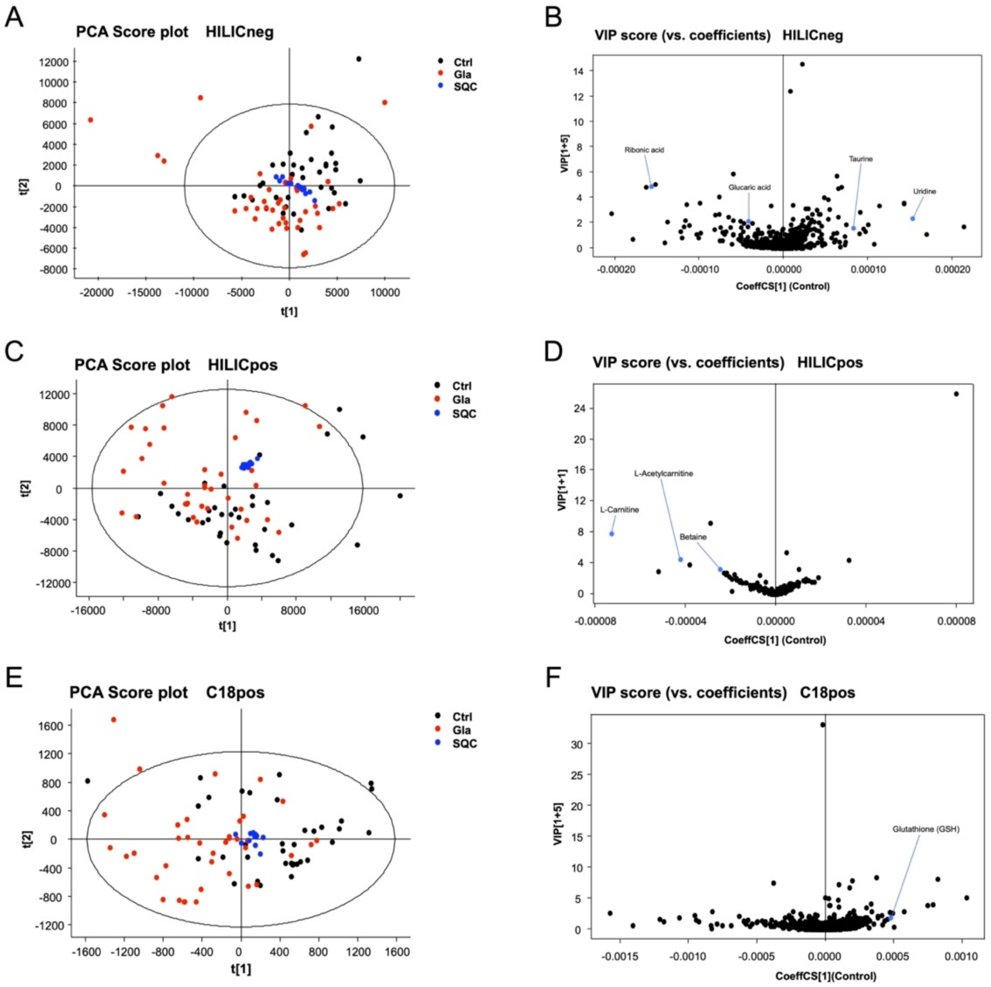

Supplementary Figure 1. PCA and VIP analysis with the HILIC negative mode (A, B), HILIC positive mode (C, D) and C18 positive mode (E, F) after removing the eye-drop component.

Supplementary Figure 2

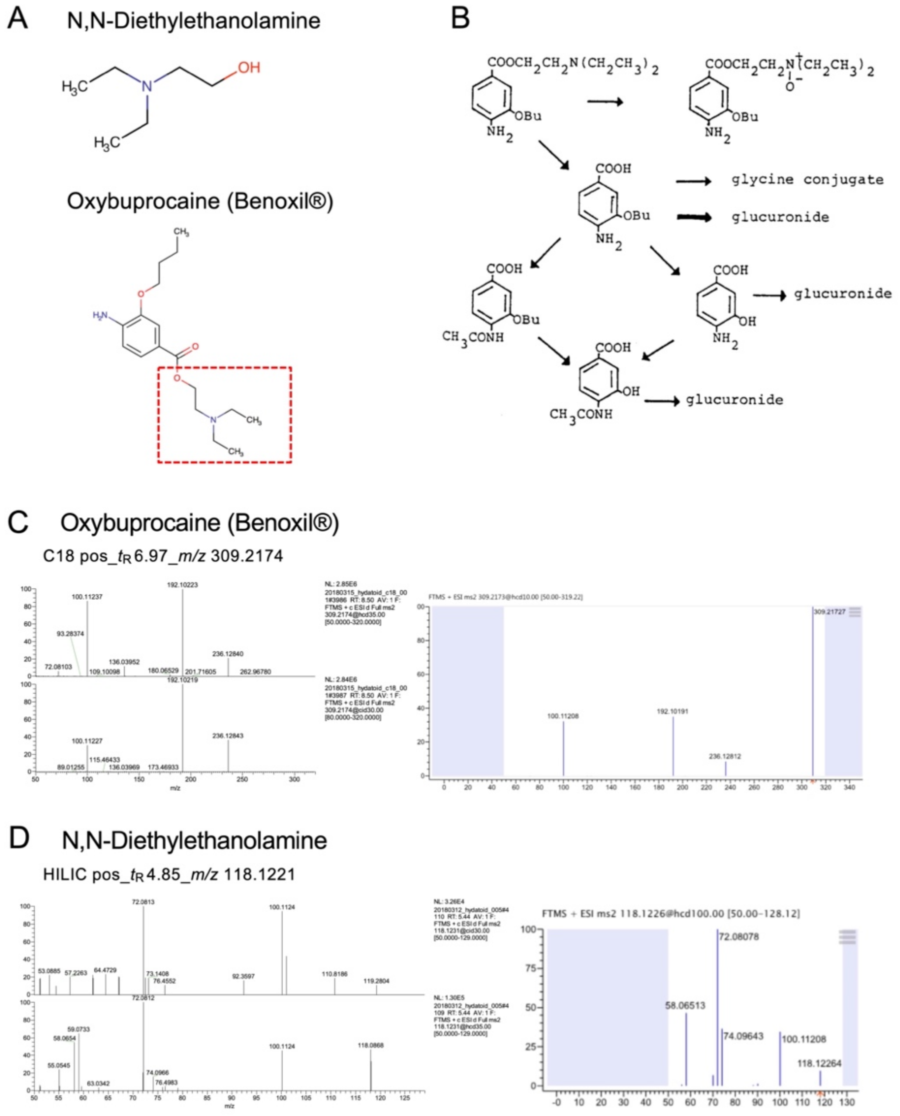

Supplementary Figure 2. N,N-Diethylethanolamine as an intermediate metabolite of oxybuprocaine. (B) The metabolic pathway of oxybuprocaine is reprinted from Journal of Pharmaceutical Sciences, 76(4), Kasuya F, Metabolism of Benoxinate in Humans, 303-305, 1987, with permission from Elsevier.

Supplementary Figure 3

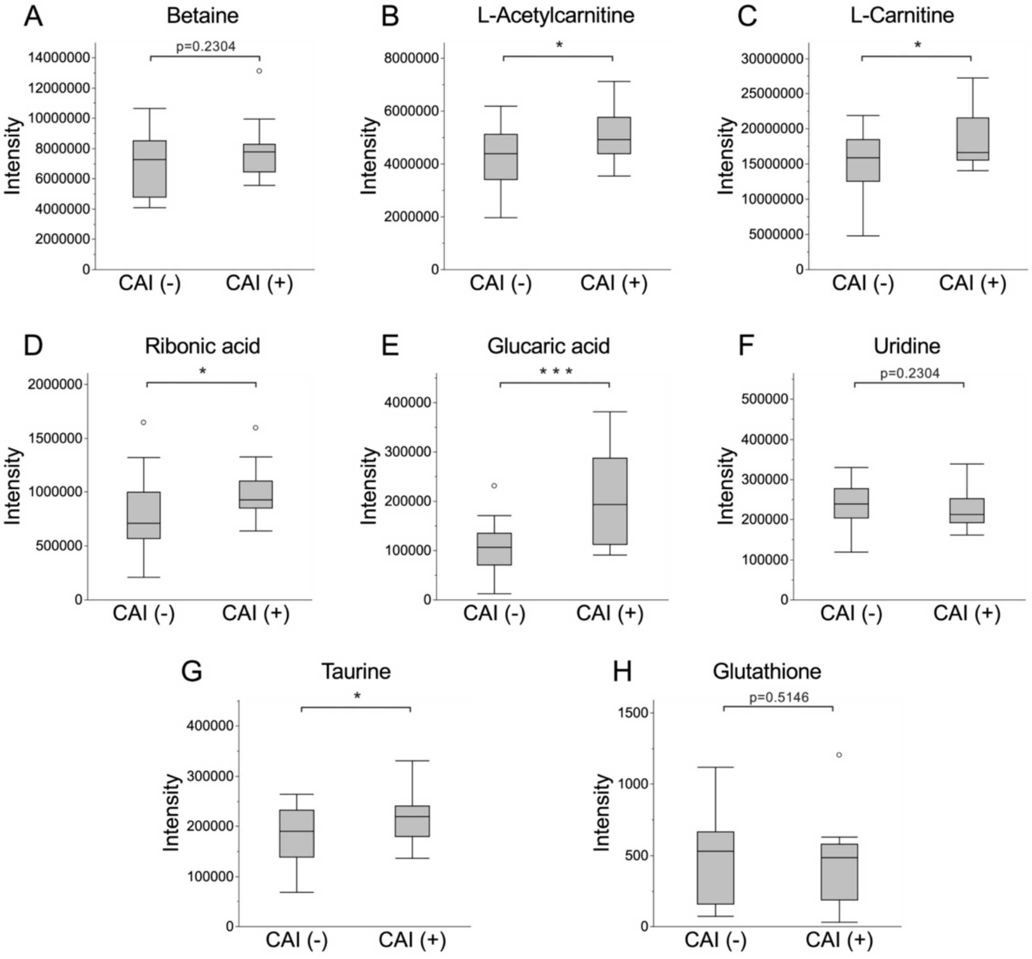

Supplementary Figure 3. Comparison of aqueous humor metabolites with and without acetazolamide in glaucoma patients. Boxplots showing the metabolite intensity of betaine (A), L-acetylcarnitine (B), L-carnitine (C), ribonic acid (D), glucaric acid (E), uridine (F), taurine (G), and glutathione (H) compared without acetazolamide (CAI-, n=25) and with acetazolamide (CAI+, n=15). This statistical analysis used the Student t-test. *P < 0.05, ***P < 0.001.

Supplementary Figure 4

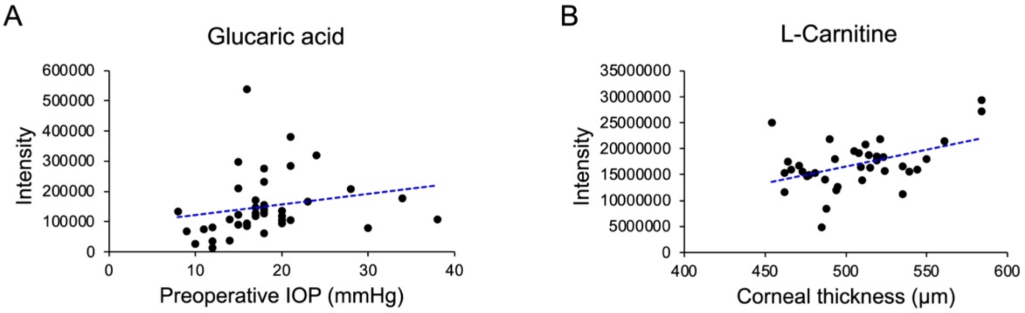

Supplementary Figure 4. Association of aqueous humor metabolites and clinical parameters in glaucoma patients. (A) Scatterplot showing the correlation of preoperative intraocular pressure and the aqueous humor level of glucaric acid. (B) Scatterplot showing the correlation of central corneal thickness and the aqueous humor level of L-carnitine.

Supplementary Figure 5

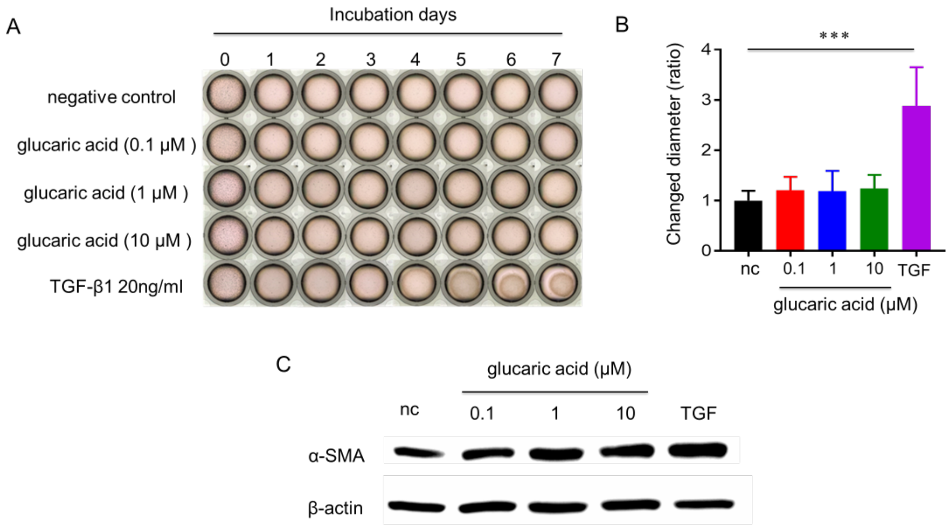

Supplementary Figure 5. Effects of glucaric acid on functional and morphological changes in human trabecular meshwork cells (HTMCs). (A) Representative photographs of collagen gel preparations of HTMCs and changes over 7 days. (B) Histogram showing changes in gel diameter, determined by comparing collagen gel surface diameter on day 7 and day 0 for HTMCs treated with glucaric acid, TGF-β1 (20 μg/m) or sterilized distilled water as a negative control (nc). Data are shown as mean ± SD (n=16 in each well) and were analyzed with Dunnett's multiple test. ***P<0.001 vs. the control group. (C) Western blot for αSMA in HTMCs treated with glucaric acid or TGF-β1 (20 ng/ml) 24 hours after treatment. ***P<0.001 vs. the control group.

Supplementary Table 1

List of compounds in glaucoma eye drops

Supplementary Table 2

Extracted feature from OPLS-DA S-plot.

Supplementary Table 3

Extracted feature from VIP.

Supplementary Table 4

Comparison of aqueous humor metabolites in glaucoma patients receiving and not receiving intraocular pressure-lowering drugs.

Supplementary Table 5

The list of MRM for targeted compounds by negative ion mode.

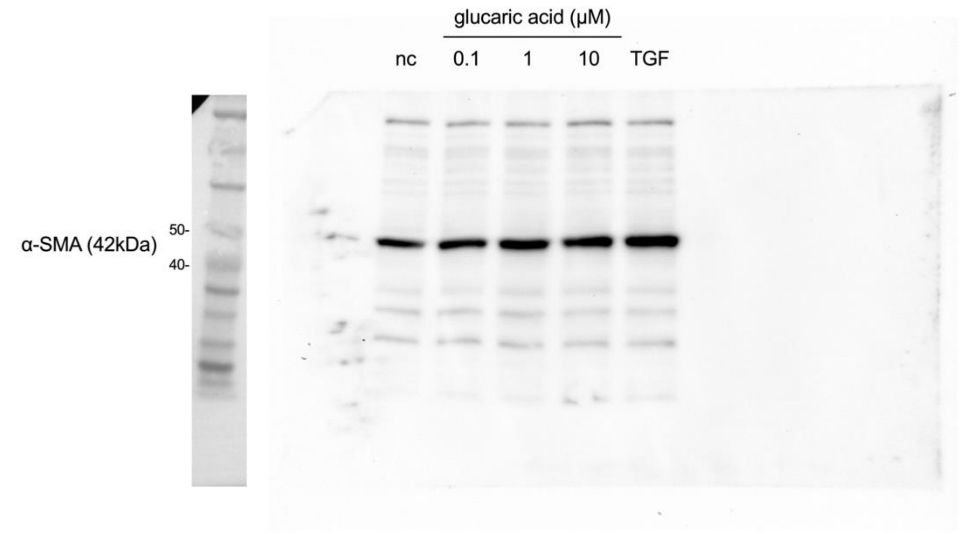
glucaric acid (μM)
nc
0.1
1
10
TGF
α-SMA (42kDa)
50-
40-

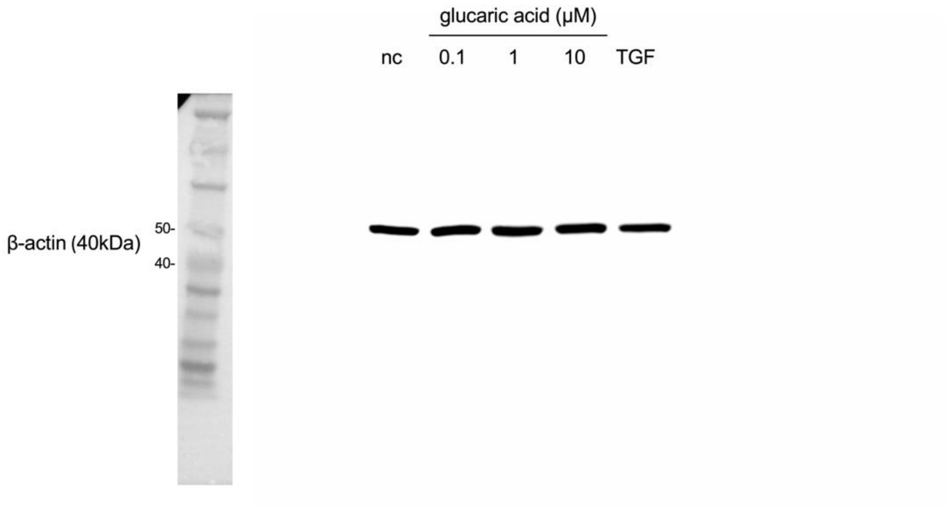
glucaric acid (μM)
nc
0.1
1
10
TGF
β-actin (40kDa)
50-
40-

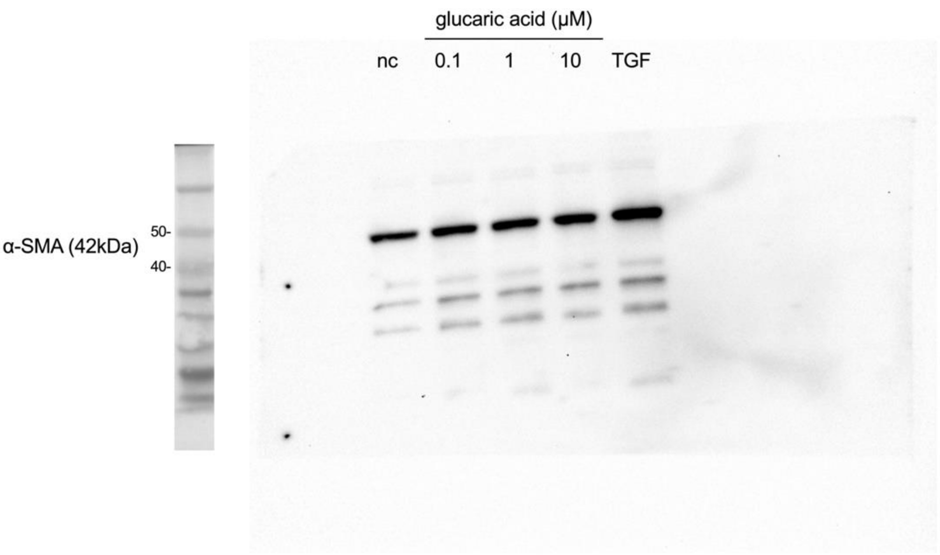
glucaric acid (μM)
nc
0.1
1
10
TGF
α-SMA (42kDa)
50-
40-

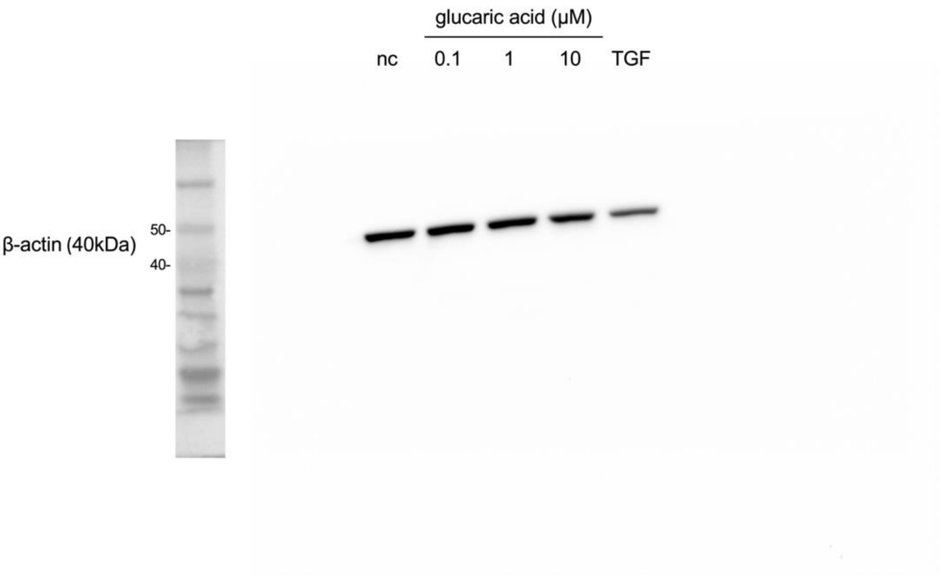
glucaric acid (μM)
nc
0.1
1
10
TGF
β-actin (40kDa)
50-
40-

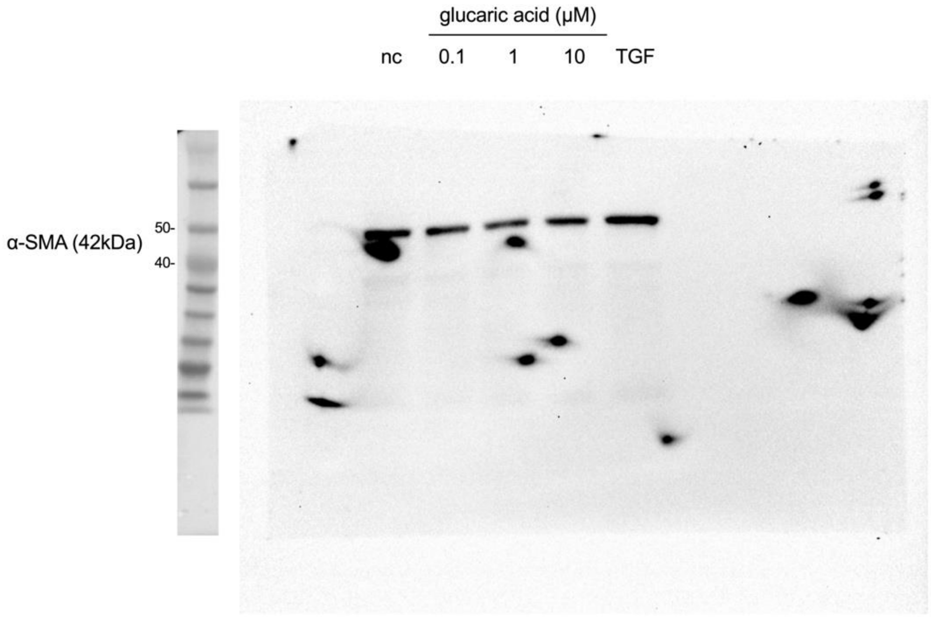
glucaric acid (μM)
nc
0.1
1
10
TGF
α-SMA (42kDa)
50-
40-

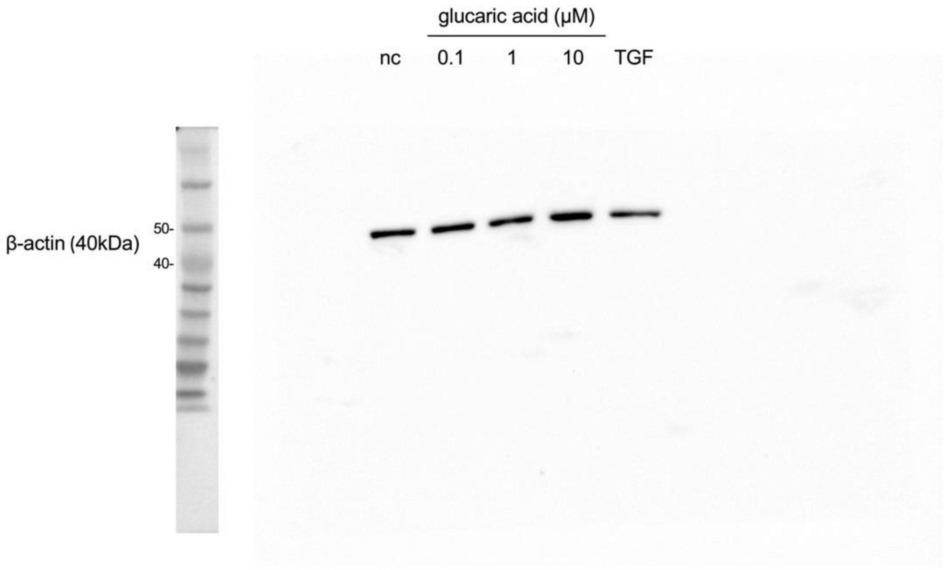
glucaric acid (μM)
nc
0.1
1
10
TGF
β-actin (40kDa)
50-
40-

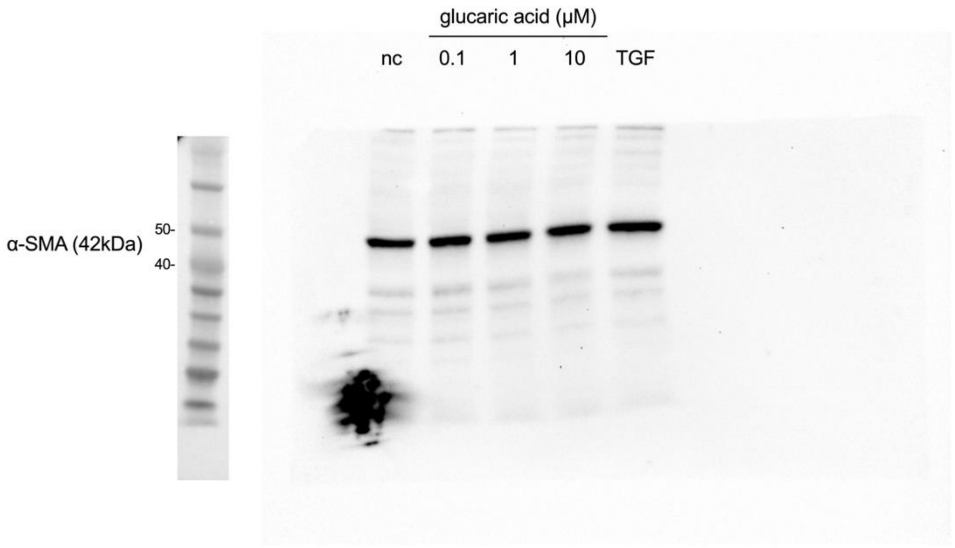
glucaric acid (μM)
nc
0.1
1
10
TGF
α-SMA (42kDa)
50-
40-

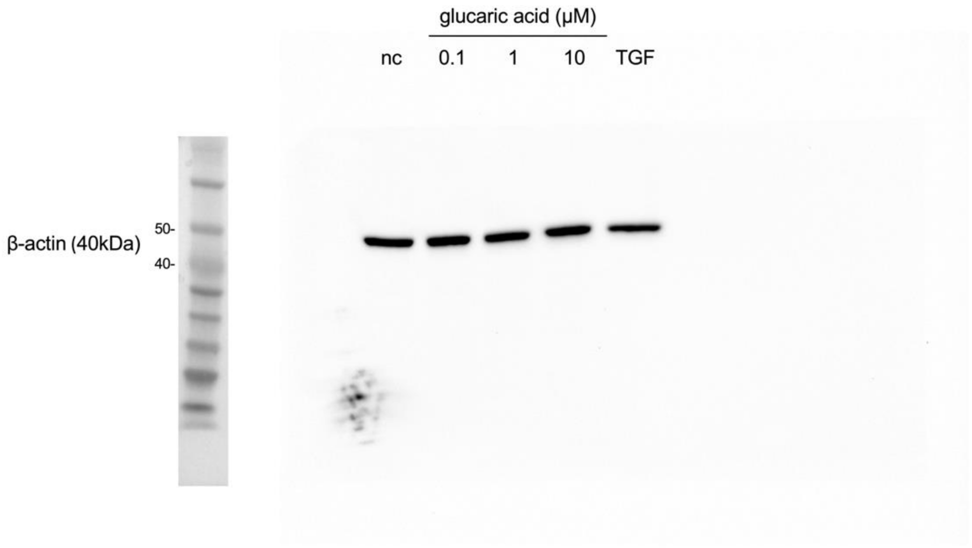
glucaric acid (μM)
nc
0.1
1
10
TGF
β-actin (40kDa)
50-
40-
